# Profiling of bacterial community associated with sugarcane rhizosphere

**DOI:** 10.1016/j.jgeb.2026.100694

**Published:** 2026-04-18

**Authors:** Himanshu Kumar, Pankaj Kumar, Malyaj R. Prajapati, Mahesh Kumar Bharti, Satya Prakash, Rekha Dixit, Kamal Khilari, Aditya Pathak, Jitender Singh, Lokesh Kumar Gangwar, Shailendra Singh Gaurav, Harshit Verma, Neelesh Kapoor

**Affiliations:** a Sardar Vallabhbhai Patel University of Agriculture and Technology, Meerut, U.P. 250110, India; b ICAR - Indian Agricultural Research Institute, Pusa, New Delhi 110012, India; c Chaudhary Charan Singh University, Meerut 250004, India

**Keywords:** Microbiome, Rhizosphere, *Saccharum officinarum*, Microbial taxa

## Abstract

Sugarcane (*Saccharum officinarum* L.) is an important cash crop in India, and its cultivation contributes significantly to the country’s economy by producing sugar, bioethanol, and other valuable by-products. The rhizosphere microbiome plays a vital role in nutrient cycling, plant health, and soil functioning. Hence, the soil microbiome associated with sugarcane cultivation plays a crucial role in nutrient cycling, plant health, and overall ecosystem functioning. However, information on the composition and diversity of bacterial communities in sugarcane rhizosphere soils of Uttar Pradesh remains limited. In different places, the outcomes of different cropping systems on the microbiome differ, affecting crop health and productivity. In the present study, bacterial diversity was characterized using 16S rRNA amplicon sequencing across different locations. The results revealed distinct variations in microbial community composition among sites, as supported by beta diversity analysis. Dominant bacterial phyla included *Pseudomonadota*, *Bacillota*, *Bacteroidota*, *Actinomycetota*, and *Acidobacteriota*, indicating their potential roles in rhizosphere functioning. These findings provide insights into the structure of sugarcane-associated microbial communities and highlight their importance in sustainable crop production.

## 1 Introduction

The rhizosphere, defined as the narrow zone of soil surrounding plant roots, is a highly dynamic environment where complex interactions occur between plants and microorganisms. These interactions play a crucial role in nutrient cycling, soil fertility, and plant health. Rhizosphere-associated microbial communities contribute to essential ecological functions such as organic matter decomposition, nitrogen fixation, and suppression of soil-borne pathogens, thereby directly influencing plant growth and productivity^1^. Until now, research has focused on the soil-associated microbiomes of various food crops, including rice, maize, soybean, grapevine, citrus, and wheat^2^. Among the various crops, sugarcane (*Saccharum officinarum*) stands out not only for its economic importance as a global commodity but also for its intricate relationship with the rhizosphere microbiome.

Sugarcane (*Saccharum* spp.) is a water-intensive crop cultivated in over 90 countries, contributing to more than 80% of global sugar production^6^. It thrives in regions with warm temperatures, abundant sunlight, and well-distributed rainfall, making countries such as Brazil, India, China, Thailand, and Australia primary producers. Sugarcane production involves a complex interplay of agronomic practices, technological advancements, and environmental considerations. The cultivation cycle typically spans 12–18 months, during which sugarcane undergoes stages of growth, maturation, and eventual harvest^7^. Beyond its role as a primary source of sucrose, sugarcane contributes significantly to global trade and economic development.

Therefore, the sugar industry has emerged as the main source of income for numerous farmers^8^. India produced approximately 370 million tons of sugar in 2019–20. Uttar Pradesh is the largest sugarcane-producing state in India, accounting for 48.46% of the total production based on 2019–20 data. Maharashtra ranks second, followed by Karnataka in third place. The other main sugarcane-producing states of India include Bihar, Haryana, Gujarat, Andhra Pradesh, and Tamil Nadu^9^, ^10^. Crops not only support millions of livelihoods but also serve as a vital commodity in various industries, including food processing, biofuels, and pharmaceuticals. Moreover, its cultivation practices have been adapted to incorporate sustainable agricultural principles, addressing challenges such as water management, soil health, and biodiversity conservation^11^.

This grass crop is mainly cultivated for its juice, which is processed to extract sucrose, although it can also be used for producing fermentation products like ethanol and acetic acid. The by-product of molasses serves as an additive in animal feed and is used to create furfural and alcohol like rum. After juice extraction, the remaining fibrous material, known as bagasse, is frequently burned to generate the steam needed to operate the sugarcane mill. Any surplus steam is then used for electricity cogeneration. Additionally, bagasse can be repurposed to manufacture fiber products such as paper and fiberboard. Transitioning conventional farms to organic management systems, a process known as transition farming, has the potential to enhance biodiversity and promote sustainable increases in food production^3^.

Understanding the structure of the rhizosphere microbiome in sugarcane soils involves unravelling the diversity, composition, and functional roles of microorganisms associated with plant roots^4^. Advances in high-throughput sequencing techniques, including metagenomics and amplicon sequencing, have transformed our capacity to study this diversity at the molecular level, uncovering previously unrecognized microbial taxa and their functional capabilities^5^. Sugarcane is primarily grown in monocultures over extended periods, resulting in lower yields caused by soil degradation, disrupted soil biology, and heightened pest and disease pressures^12^. Understanding the composition and dynamics of these microbial communities is therefore essential for developing sustainable agricultural strategies. Restoring soil biology and fertility has become essential for improving soil health, reducing yield gaps, and achieving sustainable agricultural profitability.

Consequently, substantial research has been dedicated to exploring the diversity and contributions of sugarcane rhizobacterium to enhance crop performance. Various novel plant growth-promoting rhizobacterium (PGPRs) from the sugarcane microbiome have been identified and used to enhance agricultural productivity^13^. Despite several studies on rhizosphere microbiomes, limited information is available on the composition and diversity of bacterial communities in sugarcane rhizosphere soils of Uttar Pradesh. Understanding these microbial communities is essential for improving soil health and sustainable crop productivity. Therefore, the present study aimed to characterize the composition and diversity of bacterial communities in sugarcane rhizosphere soils collected from different regions of Uttar Pradesh using 16S rRNA amplicon sequencing.

## 2 Materials and method

### 2.1 Sample collection

Rhizosphere soil samples were collected from sugarcane fields located in two major sugarcane-growing districts of Uttar Pradesh, India, namely Hapur and Shamli. In each district, five blocks were selected, and within each block five villages were chosen for sampling, resulting in a total of 50 sampling locations (2 districts × 5 blocks × 5 villages). Samples from Hapur Blocks (coordinates: N 28°45′13″, E 77°54′02″; N 28°45′10″, E 77°51′10″; N 28° 47′31″, E 77°49′09″; N 28°44′56″, E 77°50′52″; N 28°44′56″, E 77°50′54″) and samples from Shamli Blocks (coordinates: N 29°29′50″, E 77°26′27″; N 29°30′03″, E 77°26′57″; N 29°30′33″, E 77°26′57″; N 29°30′33″, E 77°27′12″; N 29°29′60″, E 77°26′58″). The Rhizosphere soil samples were collected in May 2023 during the sugarcane elongation stage following a standard root-zone sampling procedure. Initially, the top 5 cm of surface soil was carefully removed to avoid contamination from non-rhizosphere soil. Fine sugarcane roots were then excavated from a depth of 10–20 cm, which corresponds to the active root zone of the plant. The collected roots were gently shaken to remove loosely attached soil, and the soil particles tightly adhering to the root surface were considered rhizosphere soil. This rhizosphere soil was carefully collected and transferred into sterile sampling polybags, stored in ice boxes during transportation, and stored at −80°C until further use^13^.

### 2.2 Genomic DNA extraction and sequencing

A culture-independent method was employed to analyze the bacterial composition of the rhizosphere microbiome of the specified test species. The microbial DNA was extracted from rhizospheric soil samples using the Quick-DNA Fecal/Soil Microbe DNA Miniprep Kit (ZYMO, US), following the manufacturer’s protocol. DNA purity and concentration were assessed using 1% agarose gel electrophoresis. DNA samples were diluted with sterile water to a concentration of 1 ng/μL for subsequent analyses. For sequencing, equal concentrations of DNA from replicate samples within each site were pooled to generate composite samples representing each location. The composite DNA samples were then used for complete 16S rRNA gene amplification using universal bacterial primers fD1Funi 16S (5′-AGAGTTTGATCCTGGCTCAG-3′) and rP2Runi 16S (5′-ACGGCTACCTTGTTAGGACTT-3′)(Fig. S1). The 16S rRNA gene based amplicone sequencing was performed using the Illumina NovaSeq 6000 with Nextera paired-end sequencing platform at Centyle Biotech Pvt. Ltd., New Delhi, India^15^.

### 2.3 Bioinformatics data analysis

Bioinformatics analysis was performed using OmicsBox version 3.2 software available at https://www.biobam.com/omicsbox (BioBam, Valencia, Spain). Raw paired-end sequencing reads generated from sugarcane rhizosphere samples were initially subjected to quality control and preprocessing. Adapter sequences and low-quality bases were removed using Trimmomatic, and read quality was assessed using FastQC. Reads containing ambiguous nucleotides (N bases) or with an average Phred quality score below 20 were discarded to ensure high-quality downstream analysis. To eliminate potential host-derived contamination, the trimmed reads were mapped against the *Saccharum officinarum* reference genome using Bowtie2, and reads aligning to the host genome were removed. The remaining high-quality, non-host reads were retained as microbial-originated sequences for subsequent analyses.

The filtered metagenomic reads from each sampling site were pooled and assembled de novo using metaSPAdes, optimized for complex microbial communities. Open reading frames (ORFs) were predicted from the assembled contigs using Prodigal in metagenomic mode to identify putative microbial genes (metagenes). Taxonomic classification of the predicted metagenes was performed using the Kraken2 taxonomic sequence classifier, which assigns taxonomy based on exact k-mer matches against a curated reference database. Taxonomic profiles were generated at multiple hierarchical levels, including phylum, genus, and species^2^. Alpha-diversity indices, including Shannon and Simpson indices, were calculated to assess microbial diversity within samples. Beta-diversity analysis was conducted using Bray-Curtis dissimilarity matrices, and principal coordinate analysis (PCoA) was used to visualize differences in microbial community composition among sampling sites^16^, ^17^. Statistical analyses were performed to compare the relative abundance of bacterial taxa among the different sampling groups. The taxonomic profiles obtained from 16S rRNA amplicon sequencing were analyzed using the Statistical Analysis of Metagenomic Profiles (STAMP) software (version 2.1.3). White’s non-parametric *t*-test was applied to compare the mean proportions of bacterial taxa between datasets. Differences were considered statistically significant at p ≤ 0.05^19^, ^26^.

## 3 Results

### 3.1 16S rRNA sequencing and bacterial classification

High-throughput sequencing of the bacterial 16S rRNA was performed resulted in a total of 659,435 paired-end reads generated. In total, 601,072 high-quality sequences remained survived after reads screening and trimming with approx. 270 base pair sequence length. A total of 98,867 mean base pairs was detected as classified reads, out of these 98.69% were classified with kingdom Bacteria (Table 1).

**Table 1:** 16S rRNA sequencing Reads Data.

Sample	Raw reads	Clean reads	Percent in raw reads (%)	Avg Read Length	Reads Classified
1	123,309	112,087	90.9	269.5	110,446 / 98.54%
2	102,546	95,937	93.56	272.6	95,414 / 99.45%
3	103,922	94,835	91.26	269.2	93,445 / 98.53%
4	105,033	96,585	91.96	270.4	95,071 / 98.43%
5	114,443	102,808	89.83	270.8	101,779 / 99.00%
6	110,182	98,820	89.69	267.6	97,048 / 98.21%
Total	659,435	601,072	Average	270.0167	98867/98.69%

### 3.2 Rhizosphere bacterial taxonomic composition

**Fig. 1:**
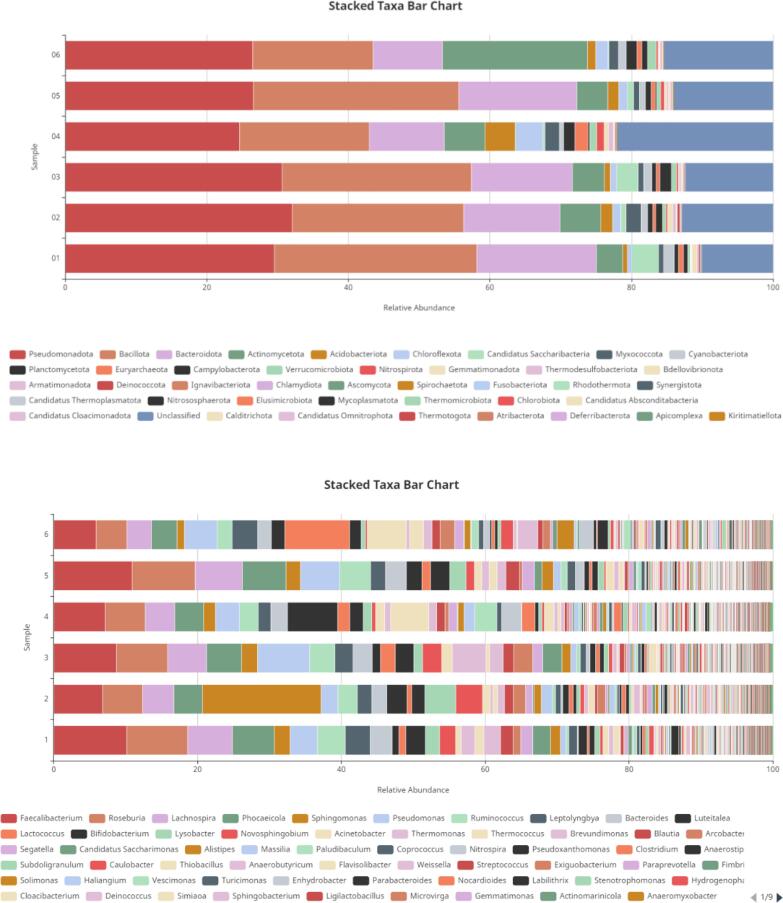
Relative abundance of major bacterial phyla and genera in the rhizosphere.

The abundance of bacterial communities was examined for all rhizosphere soil sequences at all taxonomic levels. The results were presented as taxonomic bar plots (Fig. 1) and heatmaps (Fig. 6). In all rhizosphere soil samples, more than 50 bacterial phyla were found. *Pseudomonadota, Bacillota, Bacteroidota, Saccharibacteria, Actinomycetota, Myxococcota, Acidobacteriota,* and *Planctomycetota* were the eight most abundant phyla found in sugarcane rhizospheres. Phyla such as Pseudomonadota (33.5%), *Bacillota* (30%), *Bacteroidota* (21%), *Acidobacteriota* (3%), *and Planctomycetota* (2%) were more abundant. At the genus level, the dominant genera of the sugarcane rhizosphere were *Faecalibacterium, Roseburia, Phocaeicola, Lachnospira, Sphingomonas, Pseudomonas, Ruminococcus, Leptolyngbya, Bacteroides, and Novosphingobium. Faecalibacterium, Roseburia, Phocaeicola, Lachnospira, and Sphingomonas*. At the species level, the most abundant species were *Lachnospira eligens, Roseburia hominis, Leptolyngbya* sp.*, Faecalibacterium prausnitzii, Phocaeicola dorei, Candidatus Saccharimonas aalborgensis, Phocaeicola salanitronis, Ruminococcus bicirculans, Thermomonas* sp. *HDW16, Segatella copri, Sphingomonas rhizophila, Sphingomonas xanthus, Thermococcus* sp. *M36, Paludibaculum fermentans, Phocaeicola salanitronis, Labilithrix luteola, Solimonas* sp. *K1W22B-7.*

### 3.3 Alpha diversity

**Fig. 2:**
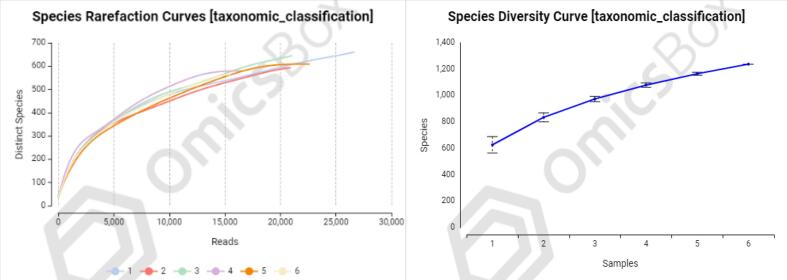
Rarefaction curve and species diversity curve showing the species richness sequence in the studied soil rhizosphere from different sites.

Alpha diversity represents the diversity of each growth phase sample by estimating the number of species in the microbial community, along with the abundance and diversity of species in the rhizospheres. This is done using a series of statistical indices, which are summarized in Table 2 (Shannon index, and Simpson index). The site 04 had the highest Shannon index (4.39), which represents the abundance and evenness of species in a community, compared to the other five sites i.e. site 01 (4.054), site 02 (4.203), site 03 (4.127), site 06 (4.389), and site 5 (4.191). The Simpson index is an indicator of species richness and evenness that is almost the same and higher at sites 06 and 04 compared to the other four sites. A rare fraction curve is an effective tool for characterizing species composition and predicting species abundance across various samples. In Fig. 2, the observed number of OTUs is plotted against the number of sequences in all six sugarcane rhizospheric soil sites. In this context, the X-axis represents the count of valid sequences filtered, while the Y-axis denotes the number of observed OTUs. Each site sample was represented by a curve of a specific color. The number of OTUs increased as the number of observed sequences increased until it reached a plateau, which indicates that the number of detected OTUs did not increase as the number of filtered sequences increased, which indicates that the sequence depth was adequate. Rarefaction curves indicate species richness and composition are higher in rhizospheric soils at sites 01 and 05 than in other sites, similar at sites 02 and 03, and lowest at sites 04 and 06.

**Table 2:** Diversity at Species Level.

Sample	Shannon Index (H) / (H / LN(N))	Simpson Index (1-D)
1	4.054 / 0.6247	0.9598
2	4.203 / 0.6579	0.9591
3	4.127 / 0.6379	09,601
4	4.39 / 0.6823	0.9681
5	4.191 / 0.6473	0.9612
6	4.191 / 0.6926	0.9739

### 3.4 Beta diversity

**Fig. 3:**
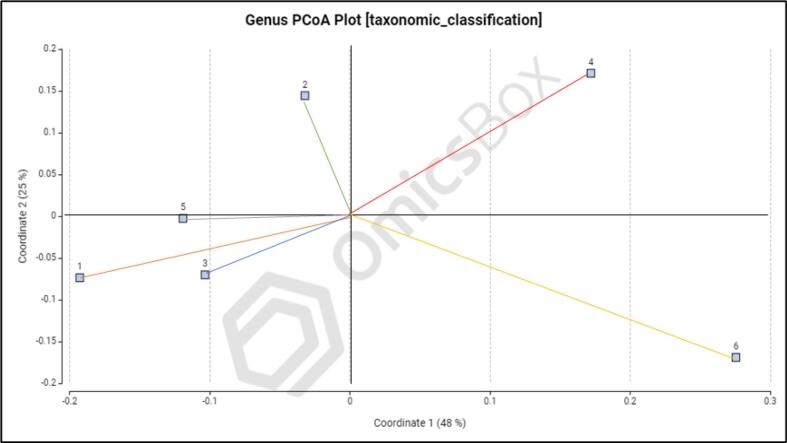
The PCoA graph of average soil microbial community genus, rhizosphere soil.

**Fig. 4:**
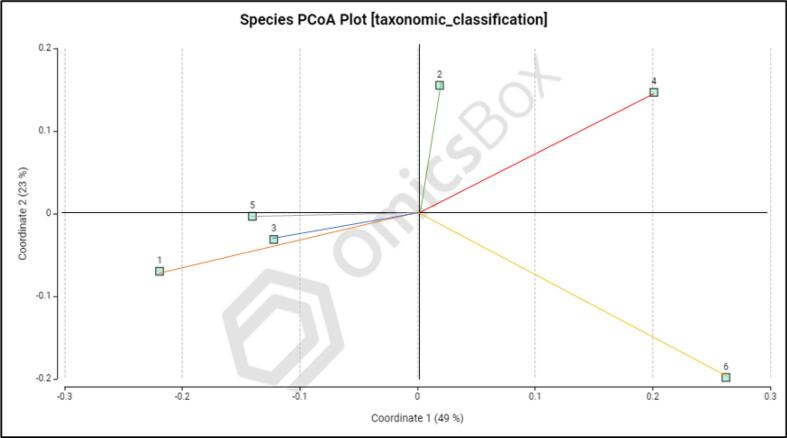
The PCoA graph of average soil microbial community species in rhizospheric soil of sugarcane.

Beta diversity analysis was performed to evaluate variations in microbial community composition across different rhizosphere samples. Principal coordinate analysis (PCoA) based on Bray-Curtis distances revealed clear separation among samples, indicating distinct microbial community structures across sampling locations (Fig. 3, Fig. 4). The first two principal coordinates explained a substantial proportion of variation (genus level: 48% and 25%; species level: 49% and 23%), suggesting that environmental or management-related factors strongly influence rhizosphere microbial composition.

The observed clustering pattern indicates that microbial communities from different sites are not randomly distributed but are shaped by site-specific soil and agroecological conditions. Such differences in beta diversity may reflect variations in soil properties, nutrient availability, and farming practices, which are known to influence microbial assemblages.

**Fig. 5:**
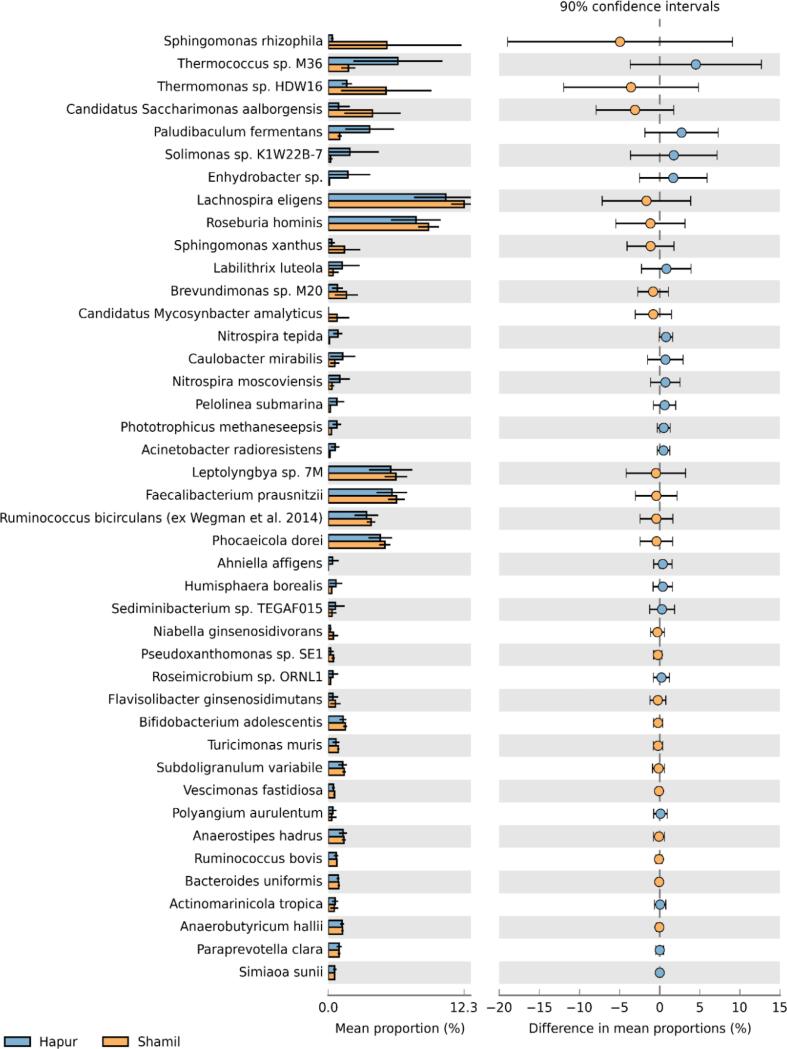
Bar plots and extended bar plots showing the main Species responsible for the significant differences between Shamli and Hapur rhizosphere soil samples investigated in the present study.

Taxonomic analysis further revealed variation in dominant bacterial phyla across locations (Fig. 5). *Bacillota* was highly abundant in Shamli soils, whereas *Pseudomonadota* dominated in Hapur samples. These groups are commonly associated with nutrient cycling and plant growth promotion, suggesting their potential role in maintaining soil fertility. The presence of diverse taxa such as *Actinomycetota*, *Acidobacteriota*, and *Myxococcota* further indicates a functionally diverse microbial community involved in organic matter decomposition and biogeochemical cycling.

**Fig. 6:**
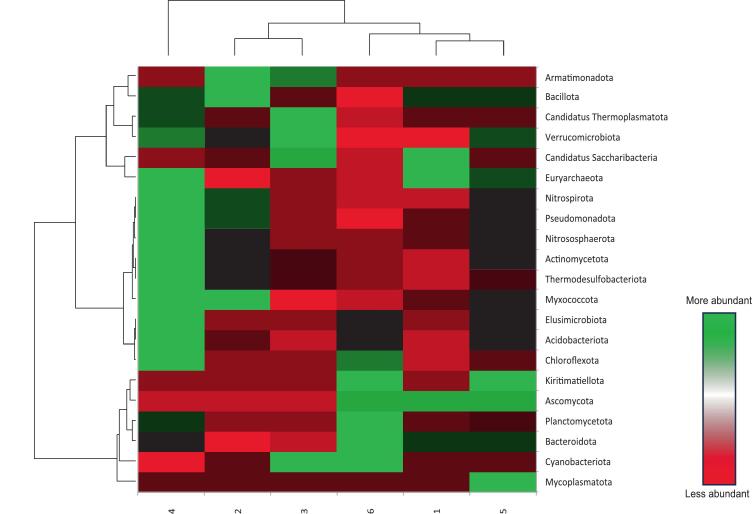
The heatmap shows the major phyla of rhizospheric soil microbial communities associated with sugarcane plant. (1-Shamli A, 2-Shamli B, 3-Shamli C, 4-Hapur A, 5-Hapur B & 6-Hapur C).

At the species level, differences in microbial composition between Shamli and Hapur soils suggest location-specific enrichment of microbial taxa, which may influence rhizosphere functionality and plant health. For example, several identified taxa are known to be involved in carbon cycling, nitrogen transformation, and plant–microbe interactions, highlighting their potential ecological significance. Overall, these findings suggest that variations in microbial community composition across sites may have important implications for soil health and sustainable sugarcane production.

## 4 Discussion

In recent years, sugarcane has remained a crop of choice across the world owing to its economic value and essential impact on humans. The microbiota associated with plants are crucial for the healthy growth and development of host plants. The rapid advancement of next-generation sequencing has brought increased attention to the microbiota in the plant rhizosphere. The microbial community structure in the sugarcane rhizosphere is shaped by a complex interaction of factors such as soil properties, plant characteristics, and environmental conditions^18^, ^19^. In previous research, Malviya et al. high-throughput sequencing was used to identify the bacterial diversity in the rhizospheres of various sugarcane species, revealing the presence of different phyla *Proteobacteria*, *Firmicutes*, *Actinobacteria*, *Acidobacter*, *Bacteroidetes*, *Chloroflexi*, *Gemmatimonadetes*, *Planctomycetes*, and *Nitrospirae* to be the most dominant species^13^. Another study revealed that the most abundant bacteria associated with sugarcane rhizospheres were *Proteobacteria, Actinobacteria, Acidobacteria, Chloroflexi, Bacteriodetes*, and *Firmicutes*^20^. The predominance of *Pseudomonadota* in several samples can be attributed to their well-known metabolic versatility and adaptability to nutrient-rich rhizosphere environments. Members of this phylum are recognized for their roles in plant growth promotion, including phytohormone production, nitrogen cycling, and suppression of soil-borne pathogens. Their higher abundance in Shamli soils may indicate favorable nutrient availability or root exudate composition supporting their proliferation^4^. This finding also roughly corresponded with those of previous studies investigating rhizospheric soil from other crops in which these phyla were examined using Illumina sequencing^2^. *Pseudomonadota* is an abundant phylum that is commonly found in soil and recognized as a plant growth promoter with heavy-metal resistance^22^, ^23^, ^24^, ^25^. The presence of *Bacteroidota* and *Acidobacteriota* further reflects functional diversity within the rhizosphere. *Bacteroidota* are known for their ability to degrade complex organic matter, indicating active carbon turnover in the soil. In contrast, *Acidobacteriota* are often associated with oligotrophic conditions and play a role in organic matter decomposition and soil carbon cycling. Their relative abundance suggests variability in nutrient availability across sampling locations^8^, ^21^. *Bacteroidetes* are the most prevalent members of the plant microbiome that suppress pathogens, playing a significant role in the rhizosphere.

The results of this study showed that analysis of alpha diversity across different growth phase samples, as represented by Shannon and Simpson indices, provides a comprehensive view of the microbial community structure in rhizosphere soil. The Shannon index, which accounts for both species’ richness and evenness, revealed site 04 as having the highest value, suggesting a high level of diversity and an even distribution of species. This was followed closely by site 06, which also demonstrated a strong diversity profile with a Shannon index of 4.389. Other sites, including site 01, site 02, site 03, and site 05, exhibited somewhat lower but still notable Shannon index values, ranging between 4.054 and 4.203^14^.

Conversely, the Simpson index, which is more sensitive to species dominance and less influenced by the number of species, indicated that site 06 and site 04 had the highest richness and evenness among the sampled sites. This aligns with the Shannon index results, suggesting that these sites not only have a diverse array of species but also maintain a balanced community structure. The rarefaction curves further elucidate these findings by providing insight into species richness and composition across different sites. The curves for sites 01 and 05 show a higher number of operational taxonomic units (OTUs) relative to the number of sequences, indicating that these sites may have a richer microbial community. Sites 02 and 03 displayed intermediate rarefaction curves, suggesting moderate richness and diversity^4^. In contrast, sites 04 and 06, despite having high Shannon and Simpson indices, showed lower OTU counts relative to the number of sequences, which could imply that while these sites have a diverse community, the number of different species detected is relatively lower compared to sites 01 and 05. These variations in microbial diversity and composition can be attributed to several ecological and environmental factors. Site 04 and site 06′s high diversity indices might be reflective of specific soil properties or environmental conditions conducive to a balanced and rich microbial community. On the other hand, the high OTU counts in sites 01 and 05 may be indicative of a broader range of microbial taxa or less environmental filtering.

## 5 Conclusion

This study provides a comprehensive assessment of bacterial diversity and community composition in the sugarcane rhizosphere across different locations in Uttar Pradesh using 16S rRNA amplicon sequencing. The results revealed significant spatial variation in microbial communities, as demonstrated by beta diversity analysis, indicating that location-specific environmental factors influence microbiome structure. Dominant bacterial phyla such as *Pseudomonadota*, *Bacillota*, and *Bacteroidota* were consistently observed, suggesting their important roles in nutrient cycling, plant growth promotion, and rhizosphere functioning*.* Thus, these findings could provide a way for promoting sugarcane health and growth by improving soil bacterial communities.

Overall, this study enhances our understanding of rhizosphere microbial dynamics in sugarcane and provides a foundation for future research aimed at exploiting beneficial microbes for sustainable crop improvement. Further studies integrating soil physicochemical properties and functional analysis of microbial communities to better understand their role in plant health and productivity.

## 6 Data availability

The sequencing reads were deposited in the Sequence Reads Archive database of the National Center for Biotechnology (BioProject Accession No.: PRJNA1147258).

## 7 Research involving animal participants

No animal experimental procedures were used in this study.

## CRediT authorship contribution statement

**Himanshu Kumar:** Writing – review & editing, Writing – original draft, Validation, Formal analysis. **Pankaj Kumar:** Writing – review & editing, Supervision, Conceptualization. **Malyaj R. Prajapati:** Writing – review & editing, Methodology. **Mahesh Kumar Bharti:** Writing – review & editing. **Satya Prakash:** Writing – review & editing. **Rekha Dixit:** Writing – review & editing. **Kamal Khilari:** Writing – review & editing. **Aditya Pathak:** Formal analysis. **Jitender Singh:** Writing – review & editing, Conceptualization. **Lokesh Kumar Gangwar:** Writing – review & editing. **Shailendra Singh Gaurav:** Writing – review & editing. **Harshit Verma:** Writing – review & editing. **Neelesh Kapoor:** Writing – review & editing.

## Informed consent

Informed consent was obtained from all individual participants included in the study.

## Funding

No funding source available.

## Declaration of Competing Interest

The authors declare the following financial interests/personal relationships which may be considered as potential competing interests: Pankaj Kumar reports financial support was provided by Sardar Vallabh Bhai Patel University of Agriculture and Technology. Pankaj Kumar reports a relationship with Sardar Vallabh Bhai Patel University of Agriculture and Technology that includes: employment. If there are other authors, they declare that they have no known competing financial interests or personal relationships that could have appeared to influence the work reported in this paper.

## Appendix A Supplementary data

Supplementary Data 1 Additional data and analyses supporting the findings of this study, including detailed experimental data.
