# Supplementary material for: Profiling of bacterial community associated with sugarcane rhizosphere
Source: J Genet Eng Biotechnol. 2026 Apr 18;24(2):100694. doi: 10.1016/j.jgeb.2026.100694 (PMC13103551; doi:10.1016/j.jgeb.2026.100694)
Supplement: Supplementary Data 1 — Additional data and analyses supporting the findings of this study, including detailed experimental data. [file mmc1.docx]

**Supplementary Material**

| **Samples** | **Pooled DNA Samples** |
| --- | --- |
| **S1** | A1, A2, A3, A4, A5, A16, A17, A18 |
| **S2** | A6, A7, A8, A9, A10, A19, A20, A21 |
| **S3** | A11, A12, A13, A14, A15, A22, A23, A24, A25 |
| **S4** | B1, B2, B3, B4, B5, B11, B12, B13 |
| **S5** | B6, B7, B8, B9, B10, B14, B15, B25 |
| **S6** | B16, B17, B18, B19, B20, B21, B22, B23, B24 |

**Table S1 Six composite sample created by pooling the DNA from each Soil sample from each field. (A- Shamli, B- Hapur)**


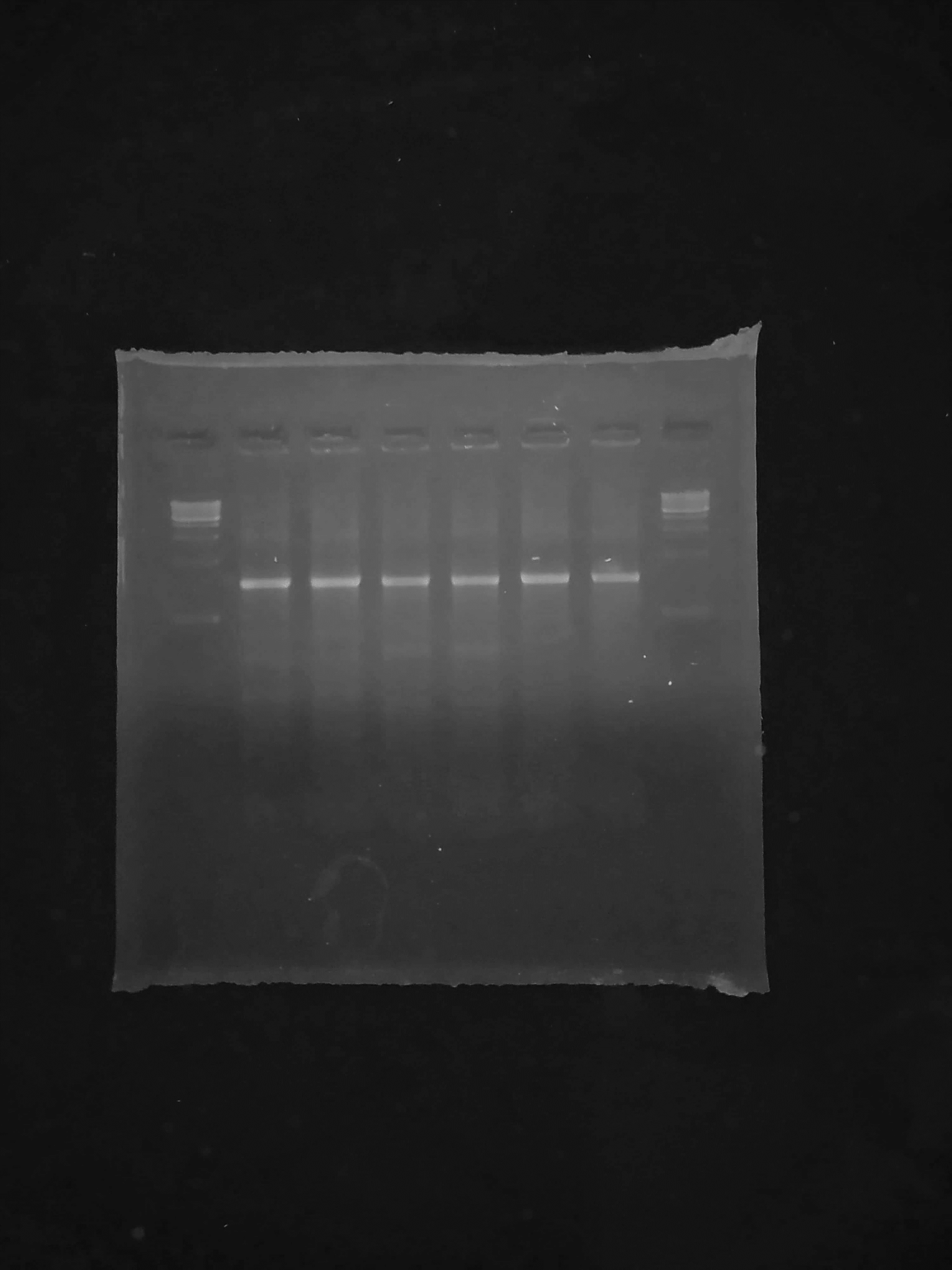


2000bp-

1000 bp-

-2000bp

-1000 bp

**Figure S1**. Agarose gel electrophoresis of amplified PCR product from 16s rRNA gene region of the DNA isolated from the rhizospheric soil.
